# Morphological, optical and photovoltaic characteristics of MoSe_2_/SiO_x_/Si heterojunctions

**DOI:** 10.1038/s41598-020-58164-7

**Published:** 2020-01-27

**Authors:** J. P. B. Silva, C. Almeida Marques, A. S. Viana, L. F. Santos, K. Gwozdz, E. Popko, J. P. Connolly, K. Veltruská, V. Matolín, O. Conde

**Affiliations:** 1 Centro de Física das Universidades do Minho e do Porto (CF-UM-UP), Campus de Gualtar, 4710-057 Braga, Portugal; 2 Departamento de Física, Faculdade de Ciências, Universidade de Lisboa, 1749-016 Lisboa, Portugal; 3 CeFEMA-Center of Physics and Engineering of Advanced Materials, Universidade de Lisboa, 1749-016 Lisboa, Portugal; 4 Centro de Química e Bioquímica e Centro de Química Estrutural, Departamento de Química e Bioquímica, Faculdade de Ciências, Universidade de Lisboa, 1749-016 Lisboa, Portugal; 5 Centro de Química Estrutural/Departamento de Engenharia Química, Instituto Superior Técnico, Universidade de Lisboa, 1096-001 Lisboa, Portugal; 6 Department of Quantum Technologies, Wroclaw University of Science and Technology, Wroclaw, 50-370 Poland; 7 GeePs, UMR CNRS 8507, (IPVF), 11 rue Joliot Curie, Plateau de Moulon, 91192 Gif sur Yvette, France; 8 Department of Surface and Plasma Science, Faculty of Mathematics and Physics, Charles University, V Holešovičkách 2, Staré Město, 18000 Prague 8 Czech Republic

**Keywords:** Two-dimensional materials, Materials for energy and catalysis, Nanoscale materials

## Abstract

This work reports the effect of different processing parameters on the structural and morphological characteristics of MoSe_2_ layers grown by chemical vapour deposition (CVD), using MoO_3_ and Se powders as solid precursors. It shows the strong dependence of the size, shape and thickness of the MoSe_2_ layers on the processing parameters. The morphology of the samples was investigated by field emission scanning electron microscopy (FESEM) and the thickness of the deposited layers was determined by atomic force microscopy (AFM). Raman and photoluminescence (PL) spectroscopies were used to confirm the high quality of the MoSe_2_ layers. Surface composition was examined by photoelectron spectroscopy (XPS). Moreover, the MoSe_2_/SiO_x_/Si heterojunctions exhibit diode behaviour, with a rectification ratio of 10, measured at ±2.0 V, which is due to the p-i-n heterojunctions formed at the p-Si/SiO_x_/MoSe_2_ interface. A photovoltaic effect was observed with a short circuit current density (*J*_*sc*_), open circuit voltage (*V*_*OC*_) and efficiency of −0.80 mA/cm^2^, 1.55 V and 0.5%, respectively. These results provide a guide for the preparation of p-i-n heterojunctions based on few-layer MoSe_2_ with improved photovoltaic response.

## Introduction

It has recently been shown that two dimensional semiconductor transition metal dichalcogenides (2D TMDs) have solved the zero-band gap drawback of graphene. They have furthermore attracted considerable attention due to their outstanding properties for various optical and photoelectrical applications^1,2^. In the past few years, Chemical Vapour Deposition (CVD) has been shown to allow the growth of large-scale monolayers of graphene^3^, boron nitride^4^ and TMDs^5–9^ of high quality, comparable to the quality of exfoliated monolayers, which makes CVD very promising regarding the fabrication of such layers on an industrial scale. Amongst the semiconductor TMDs deposited by CVD, selenides have been received less attention. This is possibly due to the lower chemical reactivity of selenium in relation to sulfur, which implies longer reaction times for large-scale deposition, inevitably leading to thicker films^10,11^. However, compared to the sulfides, monolayer MoSe_2_ has a direct band gap of 1.5 eV, close to the optimum band gap value needed for solar spectrum related applications, such as single-junction solar cells and photoelectrochemical cells^12^. Moreover, the larger spin-splitting energy of ~180 meV at the top of the valence band makes MoSe_2_ an interesting material for spintronics^9,13^.

Semiconductor TMDs have been deposited onto sapphire^14^, mica^13^ and oxidized silicon^8^ substrates. Besides its importance as a dielectric layer for the fabrication of electronic and optoelectronic devices, the presence of a SiO_x_ interlayer between the Si substrate and the TMD layers brings other advantages. The oxide layer can be easily removed by chemical means without degrading the deposited layer, enabling its suspension and transfer to other substrates (e.g. flexible substrates) as well as the integration in low dimensional devices, as 2D building blocks^15^, without the need of exfoliation methods. Furthermore, depending on the SiO_x_ layer thickness, it can produce sufficient optical contrast with respect to the deposited nanolayers, facilitating their detection by optical microscopy^16,17^.

Regarding applications, MoSe_2_ is used as an active layer in resistive random access memories (RRAMs)^18,19^ and as a complement for Cu(In,Ga)(Se,S)_2_ (CIGS) solar cells^20^, as well as in homo and heterojunctions with other TMD materials^21^. However, there are no reports concerning the photovoltaic (PV) characteristics of MoSe_2_ film in a p-i-n configuration, in spite of the fact that the integration of TMDs on Si could significantly lower the cost of photovoltaic and multifunctional devices^22^.

Here, we study the PV effect of mono- to few-layered MoSe_2_ flakes deposited by CVD onto SiO_x_/p-type Si substrates (p-i-n vertical heterostructure) and discuss in detail the PV mechanism in the heterojunctions.

## Results and Discussion

It is well known that mono-to-few layer MoSe_2_ can be grown by CVD, using MoO_3_ and Se as solid precursors, providing hydrogen is added to the carrier gas^2,9,10,23,24^. The role of H_2_ is to reduce the MoO_3_ vapour to MoO_3-x_ and enhance the reaction with Se vapour. However, the relative amount of H_2_ in the total flowing gas that leads to similar layer morphology for different CVD reactors, depends on their specific characteristics, such as the temperature profile along the CVD furnace, which in turn imposes limits on the distance between the precursors’ holders. Furthermore, also the mass ratio between the solid precursors is crucial for large-area monolayer growth^9,10,23^ due to the low chemical reactivity of Se. This mass ratio therefore needs to be optimized.

In this work we analyse the microstructure and morphology of five samples prepared with different values of H_2_% in the gas phase and of m(Se)/m(MoO_3_) mass ratio (Experimental section, Table 1).

**Table 1:** Growth parameters used in the CVD of the MoSe_2_ nanolayers.

Sample	mass (*Se*) /mass (*MoO*_*3*_)	H_2_ (%)	Total flow rate (sccm)
A	1.00	16.7	60
B	1.00	8.3	60
C	1.50	16.7	60
D	7.63	16.7	60
E	1.94	8.3	60

**Figure 1:**
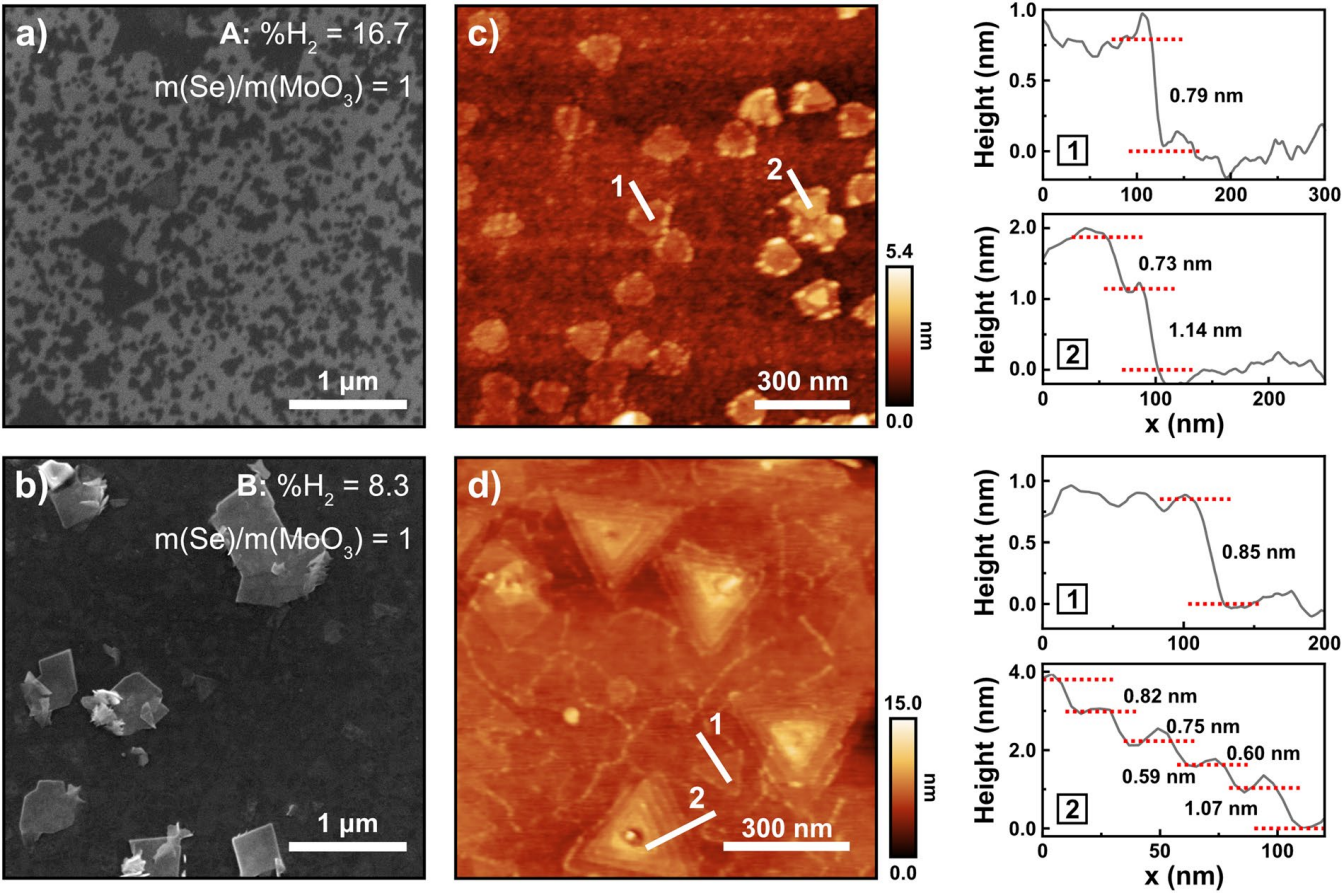
Scanning electron micrographs (**a**,**b**) of samples A and B, respectively, and their corresponding AFM images (**c**,**d**). The height profiles, taken along lines 1 and 2 on the AFM images, are shown on the right panel.

Addressing first the issue of the H_2_% in the gas phase, Fig. 1 shows the SEM micrographs of samples A (Fig. 1a) and B (Fig. 1b), and their corresponding AFM images (Fig. 1c,d), respectively), and height profiles taken along the white lines on each image. The formation of triangular flakes, which are characteristic of MoSe_2_ mono- to few-layer structures, can be observed in both samples.

However, while sample A displays the presence of triangles with an average lateral size of 154 ± 27 nm and covering about 18% of the sample’s surface area, sample B shows the triangles which are 247 ± 100 nm wide and which occupy ~31% of the surface area, resembling an almost continuous thin film.

The AFM image of sample A reveals triangles with round vertices, where some are 0.79 nm thick (profile 1), corresponding to the reported value for monolayer MoSe_2_, whereas the few-layer structures (profile 2) are indeed bilayers.

The AFM image of sample B shows multilayer triangular pyramids with lateral sizes ~320 nm, as well as smaller triangles with ~185 nm edges. In this figure, one can distinguish the different layers in each pyramid. Height profile 1 shows that the smaller triangles are monolayers of thickness 0.85 ± 0.06 nm, whereas the one measured along line 2 indicates that pyramids are 5-layer MoSe_2_ with thicknesses varying between 0.59 and 1.07 nm, showing evidence of a layer-by-layer growth process.

This H_2_% study shows that 8.3% of H_2_ in the total gas flux (sample B) allows the deposition of mono- to multilayer MoSe_2_ structures, with larger lateral sizes and covering a higher surface area. However, although the better morphology suggests a higher material quality, more multilayer structures are found in sample B.

**Figure 2:**
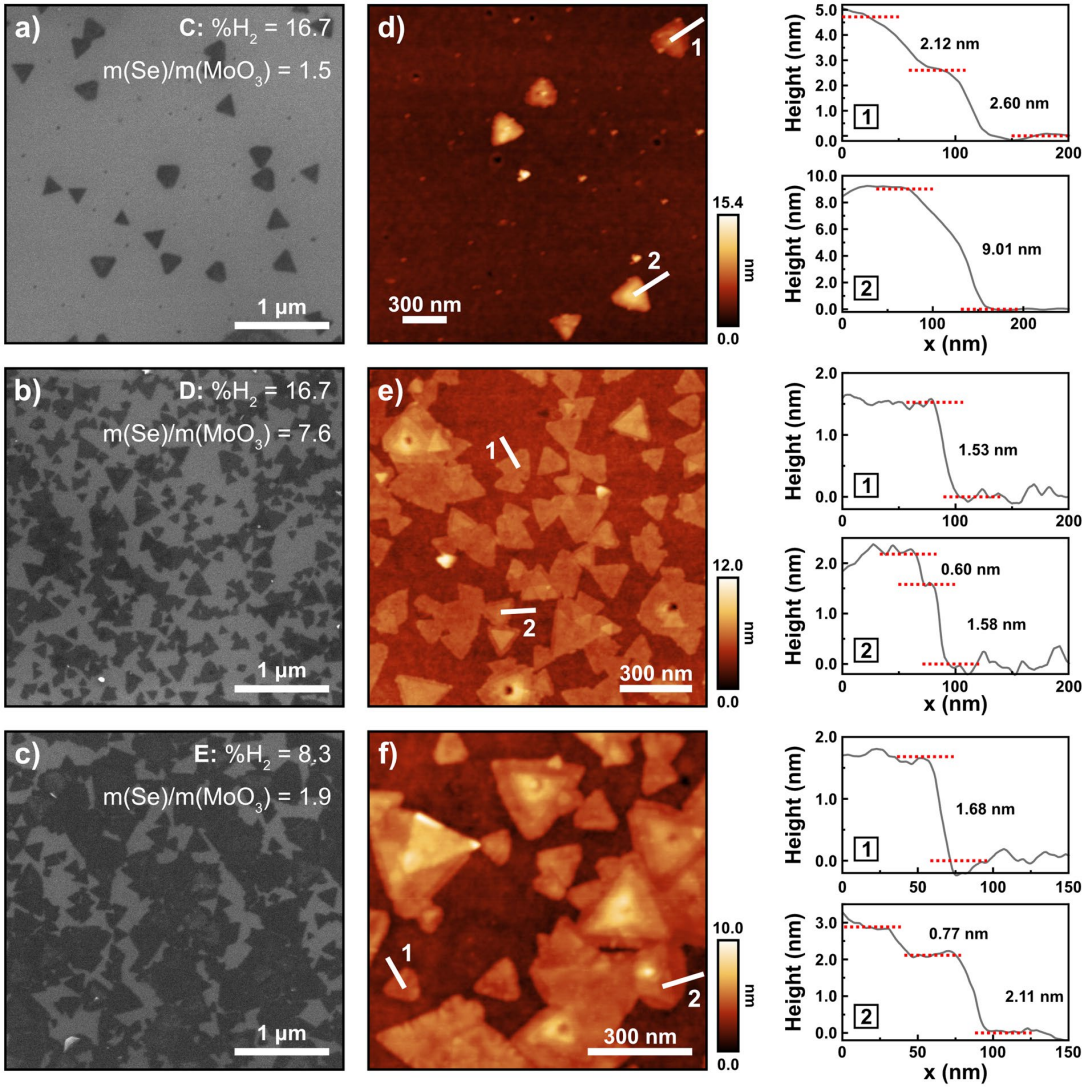
Scanning electron micrographs (**a**–**c**) of samples C, D and E, respectively, and their corresponding AFM images (**d**–**f**). The height profiles taken along lines 1 and 2 on the AFM images are shown on the right panel.

Secondly, addressing the m(Se)/m(MoO_3_) mass ratio, samples C, D and E were synthesized by increasing the m(Se)/m(MoO_3_) ratio up to ≤8 while using the previous values for the %H_2_ (Experimental section, Table 1). Figure 2 shows the SEM micrographs, AFM images and height profiles for these samples and the overall results are summarized in Table 2.

**Table 2:** Average lateral size of the triangle flakes and samples’ surface area (in %) covered by triangles, as determined by SEM.

Sample	Average lateral size (nm)	Covered surface area (%)	Number of layers
A	154 ± 27	18 ± 1	Mono- and mono/bilayer
B	247 ± 100	31 ± 3	Mono- and multilayer
C	172 ± 44	Non uniform	Tri-/4-layer and multilayer
D	168 ± 59	49 ± 2	Bi- and mono/bilayer
E	278 ± 89	57 ± 7	Bi- and mono/trilayer

The number of layers observed in the triangular structures is also presented.

In sample C, the triangles are sparsely distributed on the surface giving rise to a non uniform surface density that is as low as 3.3 ± 0.7% in some regions of the sample, while attaining a value of 15 ± 2% in others, thus comparable with the one observed in sample A; nevertheless, they have more regular shape and larger lateral sizes. The AFM analysis of this sample reveals multilayer growth. Regarding sample E, a large amount of triangles with different lateral sizes, with an average of 278 ± 89 nm, in which the larger triangles are merged with smaller ones, can be observed. This sample displays the highest value of surface area covered by triangles. Sample E, as well as D, is dominated by the presence of mono- and bi-/trilayers MoSe_2_ flakes with large sizes. These two samples were selected for further studies.

**Figure 3:**
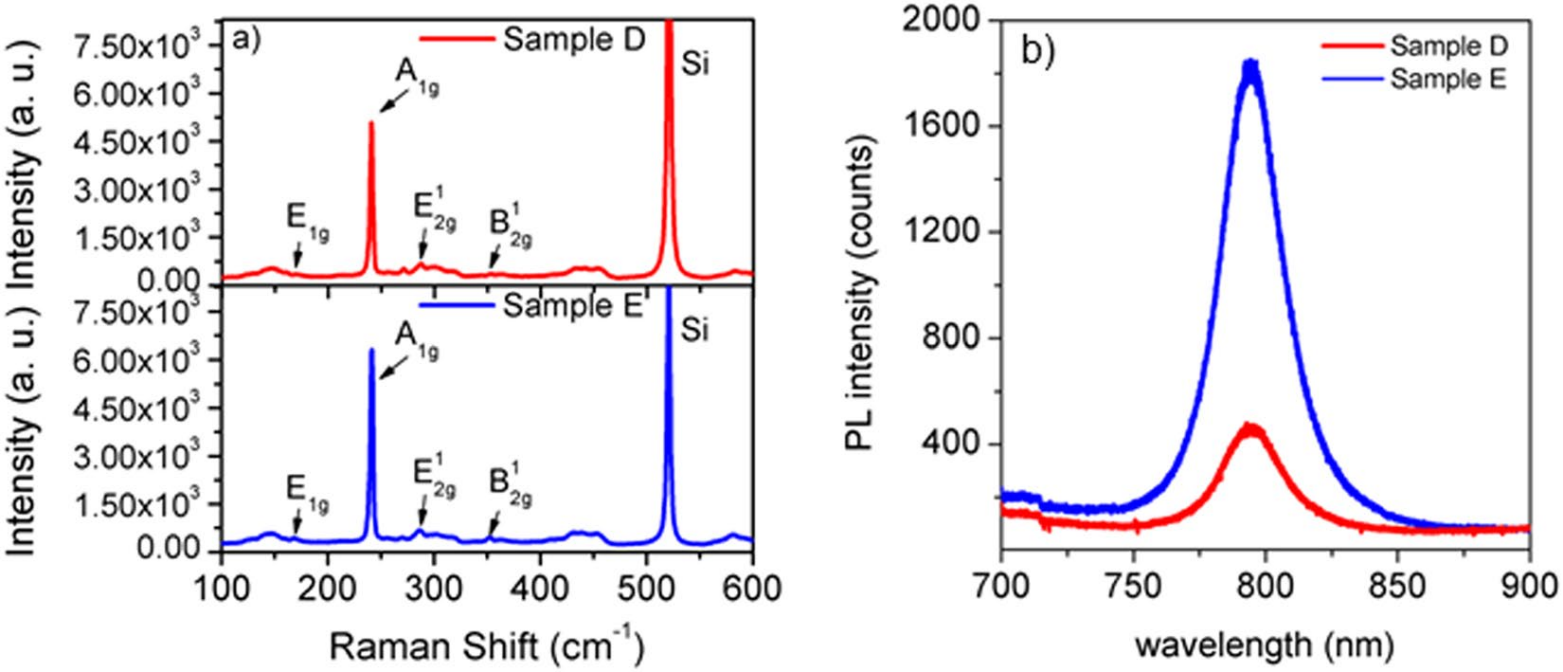
(**a**) Raman spectra of samples D and E, respectively. (**b**) Photoluminescence (PL) spectra of both samples.

Raman spectroscopy was performed to assess the structural quality of the MoSe_2_ flakes and to get a confirmation on the number of layers measured by AFM. Figure 3a shows the Raman spectra of samples D and E, respectively.

Raman spectra exhibit peaks at 161.3, 240.8, 287.3 and 352.4 cm^−1^ for sample D and 168.7, 241.4, 286.5 and 352.4 cm^−1^ for sample E. These Raman shifts correspond to the E_1g_, A_1g_, E^1^_2g_ and B^1^_2g_ modes of MoSe_2_, respectively. In particular, the A_1g_ and E^1^_2g_ modes located close to 241 and 287 cm^−1^ have been assigned to monolayer MoSe_2_^8,13,21^, along with a value of 46 ± 1 cm^−1^ for the difference Δ(E^1^_2g_ − A_1g_)^8,13,25^. Since the B^1^_2g_ mode is absent in the monolayers, the weak peak at 352.4 cm^−1^ reveals the presence of bi- and tri-layers^8,21^, in agreement with the AFM measurements.

Figure 3b displays the photoluminescence spectra of samples D and E showing a PL peak at 794 nm (1.56 eV), which is close to the reported band gap value of MoSe_2_ monolayer^25^. The fact that both samples show photoluminescence for the same wavelength is a consequence of their similarity in terms of number of layers. The difference in PL intensity between the two samples can be explained by the smaller lateral size of the triangles in sample D, which results in more grain boundaries and lower PL efficiency. Moreover, the MoSe_2_ surface coverage in sample E is higher, contributing to an enhanced PL.

**Figure 4:**
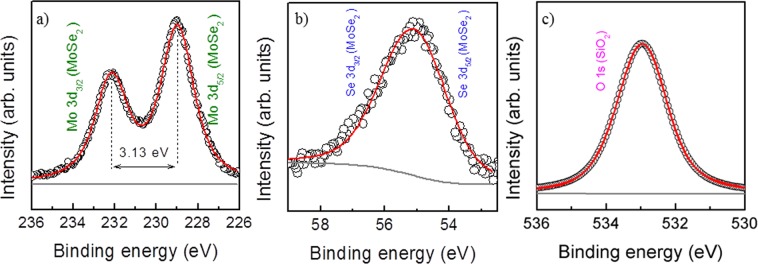
XPS spectra of (**a**) Mo, (**b**) Se and (**c**) O core levels for sample E.

Given the enhanced PL behaviour of sample E, hereafter we will focus on the properties of this sample, starting with the elemental composition and stoichiometry studied by X-ray photoelectron spectroscopy (XPS). The Mo core level XPS spectrum is shown in Fig. 4a. The peaks at 232.1 and 229.0 eV binding energies (BE) are attributed to Mo 3d_3/2_ and 3d_5/2_ core levels, corresponding to Mo^4+^ in MoSe_2_^26,27^, giving a BE difference of 3.13 eV. The Se core level XPS spectrum of sample E is shown in Fig. 4b. The peak of Se around 55 eV can be divided into Se 3d_5/2_ and Se 3d_3/2_ with peak positions at 54.6 and 55.5 eV, respectively, and a BE difference of 0.9 eV, corresponding to Se^2−^ in MoSe_2_^27^. The O 1 s spectrum (Fig. 4c) shows a single peak at 533.0 eV associated with the SiO_x_ layer from the oxidized substrate^28^, thus confirming the absence of secondary phases, such as MoO_3_ or MoO_2_. Moreover, the ∼1:2 Mo/Se ratio obtained from integrated peak areas, which were corrected by sensitivity factors, indicates that the MoSe_2_ crystals have the desired stoichiometry.

**Figure 5:**
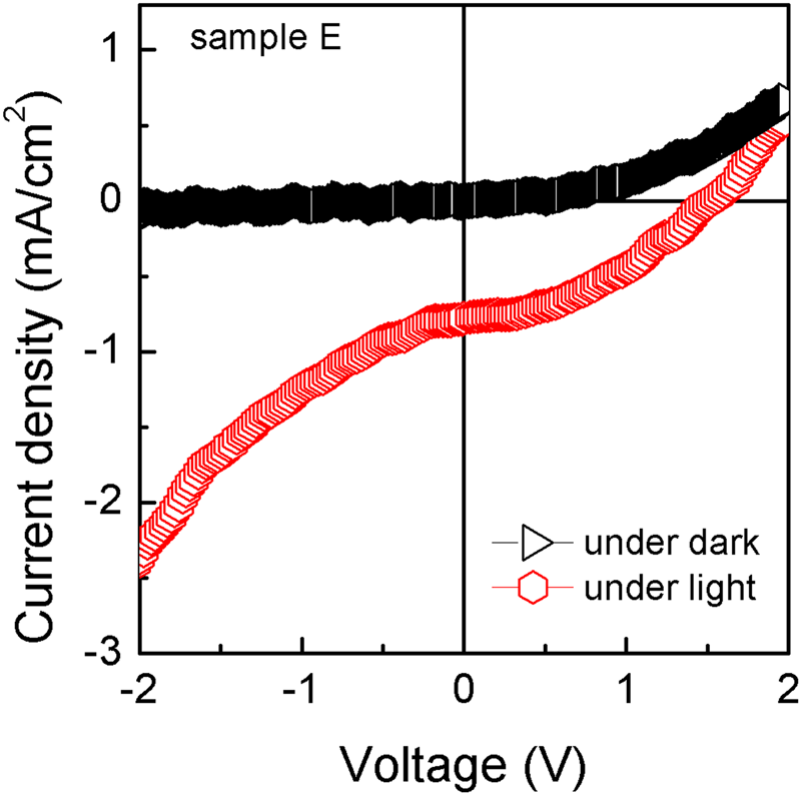
J-V curves under dark and light conditions for sample E.

The photovoltaic characteristics of the Si/SiO_x_/MoSe_2_ capacitors (sample E) were studied at room temperature and the results are plotted in Fig. 5. The black and red curves correspond to dark and light illumination conditions, respectively. J-V curves, under dark, of Al/Si/SiO_x_/MoSe_2_/ITO capacitors exhibit diode behaviour, with a rectification ratio of 10, measured at ±2.0 V. The asymmetrical behaviour of the J-V curves, under dark, indicates that the diode behaviour of the samples is due to the p-i-n heterojunctions formed at p-Si/SiO_x_/MoSe_2_ interfaces. Consequently, a built-in electrical field (E_bi_) is formed at the heterojunction. A turn-on voltage (V_on_) of 1.0 V, where the current starts to increase quickly, can be obtained. This V_on_ is higher than the one observed in MoS_2_/Si heterojunctions^22^.

From the J-V curve under light conditions, a PV effect resulting in a finite short circuit current density (*J*_*sc*_) and open circuit voltage (*V*_*oc*_) is observed. For comparison, the PV response of samples C and D, together with that from sample E, is also given in Supplementary Fig. S1 online. The *J*_*sc*_ and the V_oc_ of samples C, D and E are 0.019, −0.76, −0.80 mA/cm^2^ and −0.28, 1.30, 1.55 V, resulting in an efficiency of 0.002, 0.4 and 0.5%, respectively. The enhanced PV efficiency of sample E is associated with the different morphology including the shape, surface area covered, lateral size and thickness of the resultant MoSe_2_ layers. Moreover, due to the high quality of our heterojunctions, the *V*_*oc*_ is significantly higher than the one observed in other p-i-n and p-n heterojuntions^22,29,30^. In fact, A. U. Rehman *et al*. showed that passivation enhances the built-in field by reduction of interface trap density at the surface^31^. However, while a SiO_x_ layer is essential for good passivation of the Si surface, an excessive SiO_x_ thickness (~160 nm in our samples) can decrease the effectiveness of the carrier tunneling due to scattering and trapping of the carriers in the SiO_x_ interlayer. Therefore, the *J*_*sc*_ is not so high leading to the degradation of the PV efficiency. Nevertheless, the observed PV efficiency is similar to that observed in MoSe_2_/MoS_2_^32^ and higher than the value found in GaSe/MoSe_2_^33^ heterojunctions based transistors; it is also higher than the one in MoSe_2_ homojunctions based transistors, when measured at zero field^34^.

Different strategies are being used to enhance the PV efficiency of MoS_2_ based devices. For instance, the PV efficiency of MoS_2_/Si p-n junctions was increased from 1.3%^22^ up to 5.6% through the introduction of different passivation layers, such as SiO_2_ and Al_2_O_3_, with also different thicknesses^31,35^. Moreover, it was shown that Pd chemical doping could increase the PV efficiency of MoS_2_/Si hybrid solar cells by 375% to 2.4%^29^.

Table 3 displays a comparison of the PV efficiency obtained in the present devices and the ones found in literature for different TMD based devices with different architectures. It is worth noting again that the present PV efficiency is at least similar to the best ones found in literature for MoSe_2_ based devices. Furthermore, the outstanding *V*_*oc*_ value, which is significantly higher than the one observed in other p-i-n and p-n heterojuntions^22,29,30^, is likely to encourage further research in this area, since the *J*_*sc*_ can be further increased through a number of strategies besides decreasing the thickness of the oxide layer, such as the coupling between MoSe_2_ and plasmonic metal nanoparticles.

**Table 3:** Comparison of the photovoltaic response obtained in this work with those presented in the literature for different TMD based devices.

TMDs	Architecture	PV efficiency (%)	Reference
MoSe_2_	Vertical heterostructure	0.5	This work
MoSe_2_/MoS_2_	Transistor	≈0.5	^32^
GaSe/MoSe_2_	Transistor	0.12	^33^
MoSe_2_(thin)/ MoSe_2_ (thick)	Transistor	≈0.1	^34^
MoS_2_	Vertical heterostructure	1.3	^22^
MoS_2_	Vertical heterostructure	4.5	^35^
MoS_2_	Transistor	5.6	^31^
MoS_2_	Vertical heterostructure	2.4	^29^
MoS_2_	Vertical heterostructure	5.2	^36^
MoS_2_	Vertical heterostructure	4.6	^37^
WSe_2_ and WS_2_	Transistor	0.7	^38^

**Figure 6:**
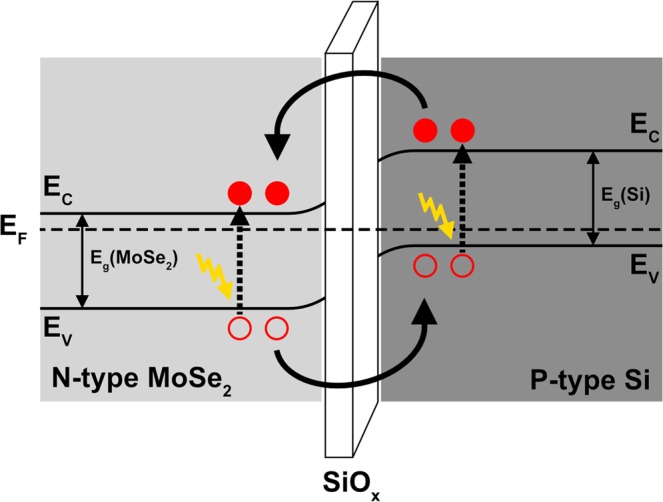
Energy band diagram of MoSe_2_/SiO_x_/Si heterojunction. E_g_ is the energy band gap, E_f_ is Fermi-energy level, E_C_ is the bottom of conduction band and E_V_ is the top of valence band.

The photovoltaic mechanism in the Al/Si/SiO_x_/MoSe_2_/ITO capacitors can be understood as follows: under light illumination, the incident photons generate the electron–hole (e–h) pairs in the MoSe_2_ film and Si, as shown in Fig. 6. The E_bi_ can effectively facilitate the separation and collection of photo-generated e–h pairs and the V_OC_ depends on the build-in potential across the interface. The processes of photo-excitation and carrier transport in the Si/SiO_x_/MoSe_2_ p-i-n junction are shown schematically in Fig. 6 showing the mechanisms responsible for photovoltaic action.

**Figure 7:**
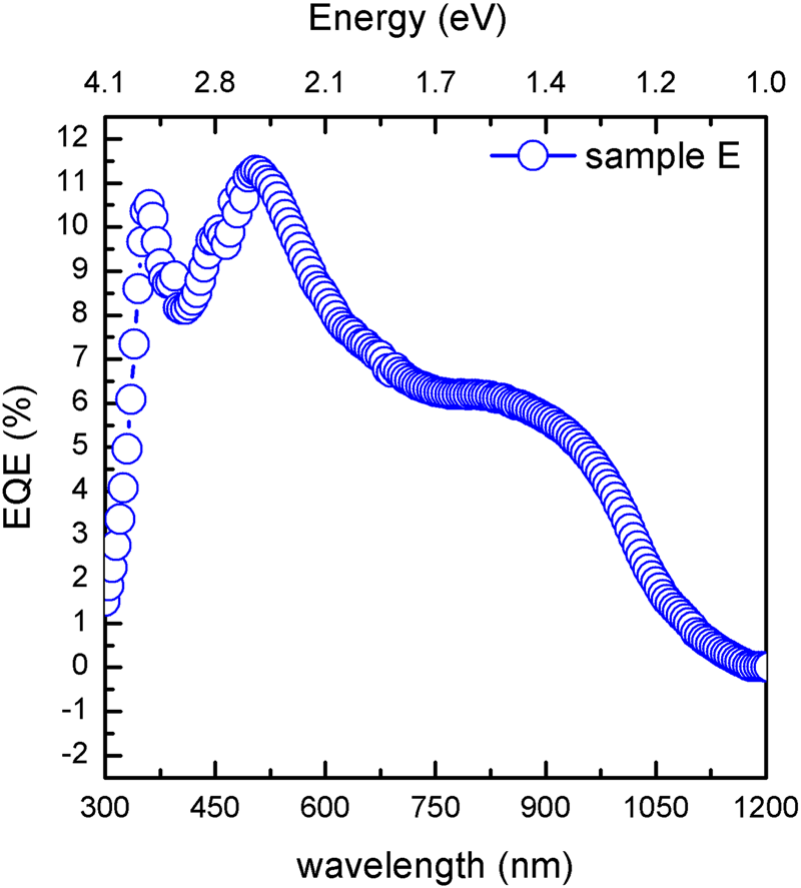
EQE as a function of wavelength for sample E.

To separate the effects of enhanced photon absorption from carrier collection, photocurrent studies under different excitation photon energies were also performed and the corresponding external quantum efficiency (EQE) was determined. Figure 7 shows the EQE as a function of wavelength obtained from photocurrent measurements for sample E. From Fig. 7, it is possible to observe that the EQE increases with increasing photon energy in the visible and then abruptly decreases in the UV. As discussed by W.J. Yu *et al*.^39^, when the EQE increases with the photon energy, one can assume that the enhanced absorbance is the primary factor responsible for the maximum EQE. For comparison, the EQE response of samples C and D together with the one from sample E is also given in Supplementary Fig. S2. The enhanced EQE of sample E can be related with the improved morphology including the shape, surface area covered, lateral size and thickness of the MoSe_2_ flakes. Moreover, the observed EQE is at least one order of magnitude higher than that found in other MoSe_2_ based devices^40^, which clearly shows that the optimization of the MoSe_2_ layers is crucial to significantly broaden the absorption spectrum, and to enhance the current conversion efficiency.

## Conclusions

This work highlights the effect of two processing parameters, as the H_2_ content and m(Se)/m(MoO_3_) ratio, on the structural, microstructural and morphological characteristics of MoSe_2_ layers. We observe that the introduction of H_2_ in the gas flow is not per se sufficient to produce high-quality MoSe_2_, and show that the ratio between the masses of precursors plays a key role in the formation of larger low dimensional MoSe_2_ layers. The microstructural, optical and elemental characteristics revealed pure and stoichiometric MoSe_2_ triangles. The photovoltaic characteristics of MoSe_2_/SiO_x_/Si heterojunctions were investigated. The PV response of the p-i-n heterojunction was evaluated and the PV efficiency is a result of the high built-in electric field developed at the heterojunction. The PV efficiency achieved is comparable to best results reported in the literature for MoSe_2_ based devices. EQE measurements also confirmed that the enhanced absorbance in few-layers MoSe_2_ is the primary factor for the maximum PV effect. Therefore, this work provides a guide to prepare few-layer MoSe_2_ onto SiO_x_/Si substrates for optoelectronic devices.

### Experimental details

Prior to the growth of MoSe_2_ layers on SiO_x_/Si substrates, the later were prepared in-house by the dry oxidation of p-doped (111) silicon wafer pieces, in a tube furnace at 930 °C and atmospheric pressure, using an oxygen flow rate of 100 sccm. The amorphous nature of the oxide layers was confirmed by X-ray diffraction (XRD) (not shown here).

The MoSe_2_ nanolayers were grown in a CVD reactor composed of a 4 cm diameter quartz tube inside a 40 cm long tube furnace. MoO_3_ powder (Neyco, 99.99% purity) and Se powder (Alfa Aesar, 99.999% purity) were loaded into quartz boats, at positions inside the reactor where their temperatures were 800 °C and 300 °C, respectively. The SiO_x_/Si substrates were placed over quartz boats with the oxide layer facing down, 1 cm away from the MoO_3_ boat at the downstream side, at a temperature of 790 °C.

The reactor was initially purged with Ar, at room temperature (RT), and then heated to 800 °C at a heating rate of 40 °C/min, keeping the Ar flux at 5 sccm. During the deposition, the Ar flux was increased and H_2_ was added into the reactor, according to the deposition parameters shown in Table 3, for the five studied samples. The reactor was kept at the growth temperature for 15 min, after which it was naturally cooled to RT, using an Ar flow rate of 30 sccm.

The surface morphology of the samples was investigated by field emission scanning electron microscopy (FESEM, JEOL 7001 F) operating in secondary electrons imaging mode and atomic force microscopy (AFM, Multimode coupled to a Nanoscope IIIa, Digital Instruments, Bruker) in tapping mode. Additionally, the surface area covered by MoSe_2_ triangle flakes and their average lateral size (Table 2) were obtained from the FESEM micrographs, by applying an intensity threshold to select the MoSe_2_ layers and using the analyse particles function of the imageJ software. AFM was used to determine the number of layers of the triangles (Table 2), by measuring their height profiles, which are the average curves of five different measurements performed along equivalent trajectories on the AFM images.

Raman microprobe spectrometry and photoluminescence (PL) measurements were performed at RT to access the quality of the MoSe_2_ flakes. For Raman spectroscopy, a LabRam HR800 Evolution (Horiba) system was used with a 532 nm excitation laser source and an 1800 grove/mm grating. The laser spot on the samples’ surface was ~1 μm^2^ and its power was ~10 mW. The excitation of the samples in the PL experiments was achieved by focusing the 514.5 nm line of an Ar^+^ laser on the samples’ surface, with a spot size less than 5 µm^2^ and an excitation power less than 100 mW to avoid any heating and nonlinear optical effects. The scattered light was analysed by using a T64000 Horiba monochromator with a 100× objective, with single grating, and a liquid-nitrogen-cooled CCD camera, in the 700–900 nm spectral range and with a typical exposure time of 240 seconds.

Chemical composition and oxidation states of the films were evaluated by X-ray photoelectron spectroscopy (XPS). XPS was performed in UHV chamber equipped with a hemispherical energy analyzer Phoibos 150. In our experiments, we used the Mg source and the binding energy scale of all XPS spectra was calibrated according to the C 1 s reference (285.0 eV).

To perform the photovoltaic characterization, indium tin oxide (ITO) top electrodes with a diameter of 1 mm were deposited by ion-beam sputtering deposition (IBSD), as described in ref. ^41^, while Aluminium electrodes were attached to the Si wafer backside by electric spark. Current–voltage (I–V) characteristics, both in the dark and under light illumination, with a maximum power density of 100 mWcm^−2^ (AM 1.5 G) were investigated. A computer controlled four-quadrant Source-Measure Unit was used to apply an external bias and measure the current. Additionally, the PV external quantum efficiency (EQE) was measured by using a Bentham PVE 300 system.

## Supplementary information

Supplementary information.
