# Supplementary material for: Morphological, optical and photovoltaic characteristics of MoSe2/SiOx/Si heterojunctions
Source: Sci Rep. 2020 Jan 27;10:1215. doi: 10.1038/s41598-020-58164-7 (PMC6985159; doi:10.1038/s41598-020-58164-7)
Supplement: Supplementary file 1 — Supplementary information. [file 41598_2020_58164_MOESM1_ESM.docx]

**Supplementary Information**

**Morphological, optical and photovoltaic characteristics of MoSe_2_/SiO_x_/Si heterojunctions**

J.P.B. Silva^a*^, C. Almeida Marques^b,c^, A.S. Viana^d^, L.F. Santos^e^, K. Gwozdz^f^, E. Popko^f^, J.P. Connolly^g^, K. Veltruská^h^, V. Matolín^h^, O. Conde^b,c**^

^a^Centro de Física das Universidades do Minho e do Porto (CF-UM-UP), Campus de Gualtar, 4710-057 Braga, Portugal

^b^Departamento de Física, Faculdade de Ciências, Universidade de Lisboa, 1749-016 Lisboa, Portugal

^c^CeFEMA-Center of Physics and Engineering of Advanced Materials, Universidade de Lisboa, 1749-016 Lisboa, Portugal

^d^Centro de Química e Bioquímica, Faculdade de Ciências, Universidade de Lisboa, 1749-016 Lisboa, Portugal

^e^Centro de Química Estrutural/Departamento de Engenharia Química, Instituto Superior Técnico, Universidade de Lisboa, 1096-001 Lisboa, Portugal

^f^Department of Quantum Technologies, Wroclaw University of Science and Technology, Wroclaw 50-370, Poland

^g^GeePs, UMR CNRS 8507, (IPVF), 11 rue Joliot Curie, Plateau de Moulon, 91192, Gif sur Yvette, France

^h^Department of Surface and Plasma Science, Faculty of Mathematics and Physics, Charles University, V Holešovičkách 2, 18000 Prague 8, Czech Republic

*E-mail: [josesilva@fisica.uminho.pt](mailto:josesilva@fisica.uminho.pt) ; [omconde@ciencias.ulisboa.pt](mailto:omconde@ciencias.ulisboa.pt)


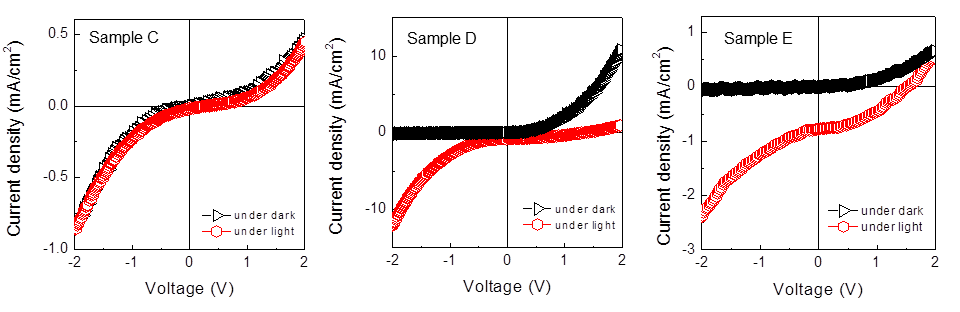


**Figure S1.** J-V curves under dark and light conditions of samples C, D and E.


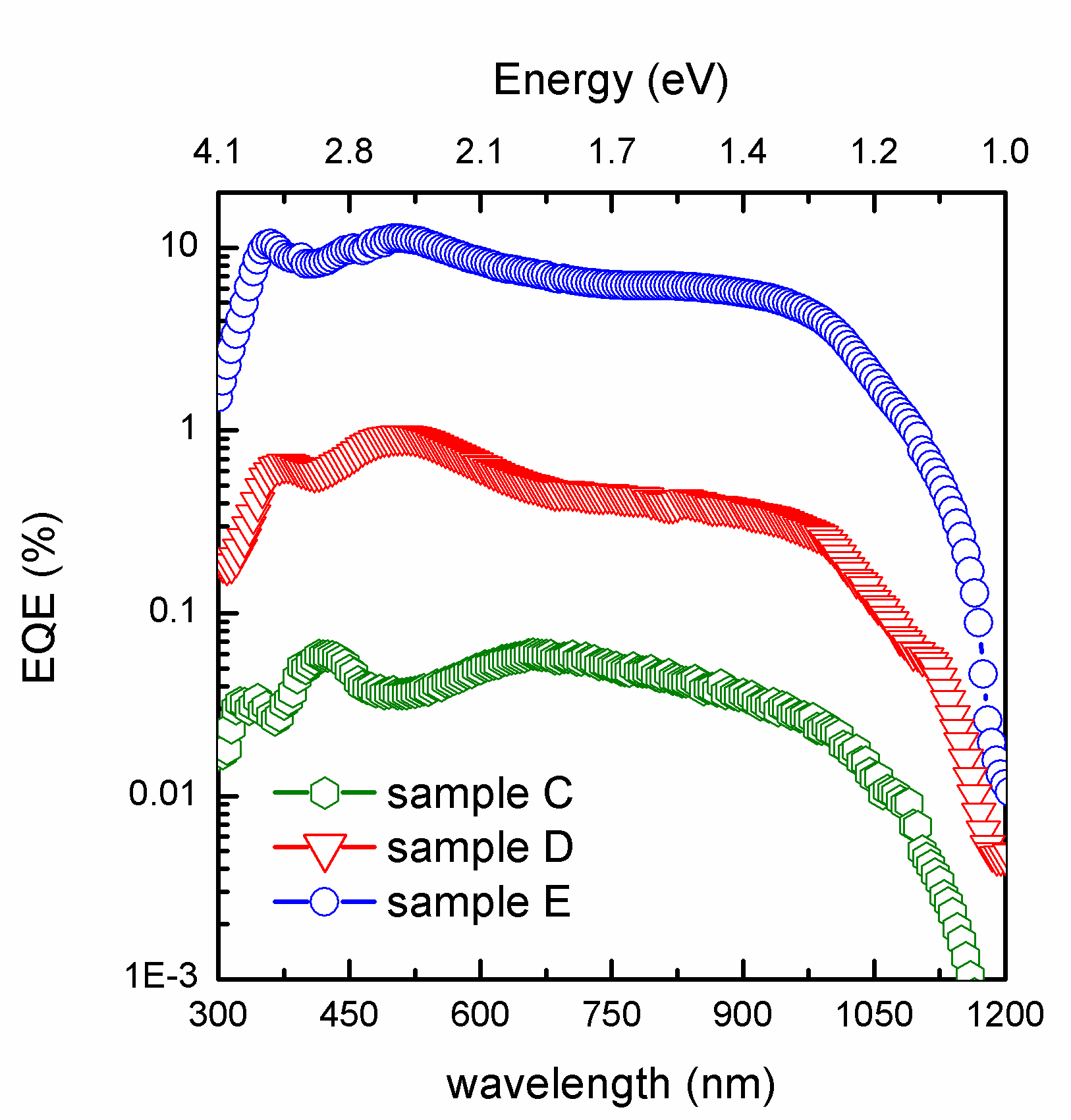


**Figure S2.** EQE as a function of wavelength for samples C, D and E.
